# Adverse Health Effects and Unhealthy Behaviors among Medical Students Using Facebook

**DOI:** 10.1155/2013/465161

**Published:** 2013-12-28

**Authors:** Sami Abdo Radman Al-Dubai, Kurubaran Ganasegeran, Mustafa Ahmed Mahdi Al-Shagga, Hematram Yadav, John T. Arokiasamy

**Affiliations:** ^1^Department of Community Medicine, International Medical University (IMU), No. 126 Jalan Jalil Perkasa 19, 57000 Bukit Jalil, Kuala Lumpur, Malaysia; ^2^International Medical School, Management and Science University (MSU), University Drive, Off Persiaran Olahraga, Section 13, 40100 Shah Alam, Selangor, Malaysia; ^3^Newcastle University Medicine Malaysia, Main Campus, Jalan Sarjana 1, Kota Ilmu, Educity@Iskandar, 79200 Nusajaya, Johor, Malaysia

## Abstract

Little is known about the relationships between adverse health effects and unhealthy behaviors among medical students using Facebook. The aim of this study was to determine the associations between adverse health effects and unhealthy behaviors with Facebook use. A cross-sectional study was conducted in a private university in Malaysia among 316 medical students. A self-administered questionnaire was used. It included questions on sociodemographics, pattern of Facebook use, social relationship, unhealthy behaviors, and health effects. Mean age was 20.5 (±2.7) years. All students had a Facebook account. The average daily Facebook surfing hours were 2.5 (±1.7). Significant associations were found between average hours of Facebook surfing and the following factors: isolation from family members and community, refusing to answer calls, musculoskeletal pain, headache, and eye irritation (*P* < 0.005). The average hours spent on Facebook were significantly associated with holding urination and defecation while online, surfing Facebook until midnight, and postponing, forgetting, or skipping meals (*P* < 0.005). The average hours spent on Facebook were associated with adverse health effects and unhealthy behaviors among medical students, as well as social isolation from the family and community.

## 1. Introduction

Facebook is by far the biggest social network worldwide. Until December 2012, Facebook had one billion monthly active users [1]. The adoption of Facebook among students increased over time. A recent study from the US showed that up to 96% of medical students regularly use Facebook [1]. The use of social networking websites is increasing extensively in the field of medical education and has gained substantial interest among educators and institutions [2]. Institutions of higher education around the world began to focus on the benefits of Facebook for educational purposes [3].

Most of the literature has focused on the potential of Facebook as a learning tool in higher education. Facebook was reported to be useful for students in the social and the academic aspects as it allows for interactive learning between students and facilitators in higher educational institutions. [4]. Like any new phenomena in our life, facebooking might hold its own risks. However, limited literatures focused on its harmful effects on health and social life. Possible impacts of Facebook use reported in the previous literature include addiction, waste of time, and money [5, 6]. Some criminal offences and deterioration of morale prompting disciplinary actions were attributed to misuse of social networking in circumstances of disclosing privacy issues and posting unethical materials [7]. Unprofessional behavior and inappropriate material posting on social networking sites have also been reported among health care providers and university staff [7].

Recent studies that focused on the impact of facebooking on mental health found association between social networking and changes in self-esteem [8], sleep disorders, and high level of depression among students [9]. Little is known about the relationships between adverse health effects, social isolation, and unhealthy behaviors and Facebook surfing hours among medical students.

This study aimed to assess the direct association between Facebook surfing and physical health, unhealthy behavior, and the social relations of medical students. This study addressed the following question: is there a relationship between the duration of facebooking (in hours) and the physical health of students and their health related behaviors as well as their social relationship?

## 2. Methods 

A cross-sectional study was conducted among 316 medical students in a private university in Malaysia where all students were asked to participate. Permission for access to the students was obtained from the coordinators and lecturers. Confidentiality and freedom to participate were assured. A written consent was obtained from the participants. The ethics committee of the university approved the study protocol. The questionnaire was pilot tested on ten students who were not participating in the study.

### 2.1. Instruments

A self-administered questionnaire was distributed to the participants. The first part included questions on sociodemographics such as age, gender, race, and year of study. The second part included questions on pattern of Facebook use such as the average daily hours of facebooking and place of Facebook use. The third part assessed the health related behavior during facebooking and it included five items: holding urine, holding defecation, postponing, forgetting, or skipping meal, and facebooking until midnight. The answer responses were “not at all,” “sometimes,” or “frequently.” The fourth part assessed perceived isolation from family and society during facebooking activities. It included three questions: during facebooking, do you (1) feel isolated from the family, (2) feel isolated from the society, (3) refuse to answer calls? The answer responses were “not at all,” “sometimes,” or “frequently.” The fifth part included questions on adverse physical health effects during facebooking; in the last month, how frequently did you experience the following conditions during Facebook use: back pain, shoulder/neck pain, wrist joint pain, headache, and eye irritation? The response options were “not at all, sometimes, or frequently.”

### 2.2. Statistical Analysis

The Statistical Package of Social Sciences (SPSS) (version 16.0) was used for data analysis in this study. Normal distribution: normality test of the number of facebooking hours/day yielded abnormal distribution. Nonparametric Mann-Whitney *U* test was used to assess the association between facebooking hours and other variables. To ease interpretation, we categorized “not at all” and “sometimes” as “no” and “frequently” as “yes.” The accepted level of significance was set below 0.05 (*P* < 0.05).

## 3. Results 

Three hundred out of 316 medical students returned valid questionnaires with a response rate of 94.9%. The majority of respondents were females (68.0%), were Malay (63.7%), and were currently in their first year of study (42.3%) (Table 1). All respondents had a Facebook account. The mean (±SD) of daily Facebook surfing hours was 2.5 (±1.7) hours and the total surfing hours ranged from one to eight hours per day. Most respondents surfed Facebook at their homes (67.0%), particularly in their bedroom (65.7%) followed by surfing Facebook in their living room (27.7%) or dining room (5.7%), respectively. Eighty-one students (27.0%) reported to surf Facebook through mobile gadgets (Table 2).

Table 3 exhibits the adverse health effects of Facebook usage among medical students. Two hundred nine students complained of occasional back pain, while another one hundred ninety-five of them had occasional shoulder pain. Additionally, the majority of respondents experienced occasional wrist pain, headaches, and eye irritations during Facebook surfing.  Table 4 shows respondents' health related behaviors during Facebook surfing. The majority of respondents denied holding urine and defecation or skipping meals during Facebook surfing. However, most of the respondents agreed to postponing meals occasionally during Facebook usage. Two hundred two respondents had occasional midnight logins for facebooking activities.

Table 5 exhibits intrapersonal factors of Facebook surfing among respondents. The majority of respondents denied feeling isolated from family (63.7%) and society (61.0%) while facebooking. However, most respondents (65.7%) prefer surfing the Facebook in their own bedrooms. The majority of respondents denied refusing to answer calls during facebooking activities (56.0%).

There was a significant association between back pain, shoulder pain, and neck pain and Facebook surfing hours (*P* < 0.01). Headaches and eye irritations also showed significant associations with Facebook surfing hours (*P* = 0.009 and *P* = 0.008, resp.) (Table 6).

There was a significant association between holding urine and defecation with facebooking activities (*P* < 0.001). Postponing or skipping meals and midnight logins also showed significant associations with facebooking activities (*P* < 0.001) (Table 7). Significant associations were found between feeling isolated from the family and the society with facebooking activities (*P* < 0.001, *P* = 0.008, resp.) (Table 8).

## 4. Discussion 

This study found preliminary associations between behavioral and intrapersonal factors affecting Facebook surfing hours and health well-being among medical students in Malaysia. The year 1995 marked the inception of internet into medical education and university students [10].

Our sample showed that the majority of students surfed Facebook between one to eight hours a day, inconsistent with the findings reported by Kalpidou et al. [11] who found that the majority of university students surfed between one to two hours per day only. Some researchers argued that students mainly use the social network to keep in touch with other individuals, with minimal informal academic use of Facebook for learning purposes [12]. Raacke and Bond-Raacke [13] found that only 10.9% of students use Facebook for academic purposes. The adoption of Facebook as a channel of learning resources by medical students is fundamental to cope with the paradigm shift towards self-directed learning in medical schools globally. Thus, the time of medical students (as an important resource) is better to be used mainly for academic purposes rather than for unnecessary activities.

The dawn of social networking in the digital era had marked the beginning of cyberpsychology, causing adverse physical and mental health effects to human well-being [14, 15]. This study found significant associations between Facebook surfing hours with potential adverse health effects among medical students. Medical students surfing Facebook had significantly experienced musculoskeletal pain (back pain, shoulder pain, or neck pain), eye irritation, and headaches.

This study found significant associations between Facebook surfing hours and unhealthy behavioral actions like holding urine and defecation, postponing or skipping meals, and facebooking until midnight. Young [14] found that university student's sleep patterns were disrupted due to late night logins and surfing, causing extensive sleep deprivation side effects like excessive fatigue, impaired academic performance, and decreased immunity. Sharifah et al. [15] identified the behavioral consequences of social networking in the current digital era as hyperactivity, inattention, depression, and multitasking mania.

This study is the first to enumerate significant associations between intrapersonal factors (social isolation from family and society and self-isolation) with Facebook surfing. These findings were consistent with the Gratification Theory interpreted by Cabral [16] that motivation related factors to social networking are influenced by social escapism, pass time, interactive control, information, and communication. Facebook exhibited an ultimate isolating technology among tech savvy youngsters and indeed reduced their participation and interconnectivity activities in many real-life situations in the world [15].

The cross-sectional design of this study cannot prove a causal relationship between the variables. The study sample was exclusively made up of students from a single university. This may affect the generalizability of the findings from this study.

In conclusion, this study found a significant association between Facebook use and many adverse health effects and unhealthy behavior. This study conveys a message to both students and educators that Facebook might have some negative issues not without its issue.

Health education and promotion should be proposed by educational institutions to create awareness of the possible health adverse effect of Facebook. Safety precautions should be considered during its use. Health screening of students for musculoskeletal disorders and eye disorders periodically is recommended. Self-control of Facebook surfing hours is essential to avoid the possible health consequences. Future research is recommended to assess the costs and benefits of using Facebook. Severity and frequency of musculoskeletal disorders and other health effects should be investigated in future studies.

## Figures and Tables

**Table 1:** Sociodemographic characteristics of respondents (*N* = 300).

Characteristics	*N*	Percentage (%)
Gender		
Males	96	32
Females	204	68
Race		
Malay	191	63.7
Chinese	29	9.7
Indians	61	20.3
Others	19	6.3
Year of study		
1	127	42.3
2	69	23.0
3	74	24.7
4	30	10.0

**Table 2:** Location of Facebook use among respondents (*N* = 300).

Location of Facebook use	*N*	Percentage (%)
Home	201	67
School	5	1.7
Cafe	3	1.0
Library	1	0.3
Mobile	81	27
Friends' house	9	3.0
Room		
Living room	83	27.7
Bedroom	197	65.7
Dining room	17	5.7
Others	3	1.0

**Table 3:** Adverse health effects of Facebook usage among respondents (*N* = 300).

Health effects	Frequency	*N*
Back pain	Never	74
	Sometimes	209
	Frequently	17
Shoulder pain	Never	76
	Sometimes	195
	Frequently	29
Wrist pain	Never	103
	Sometimes	185
	Frequently	12
Headache	Never	93
	Sometimes	187
	Frequently	20
Eye irritation	Never	80
	Sometimes	193
	Frequently	27

**Table 4:** Health related behaviors during Facebook surfing (*N* = 300).

Behaviours	Frequency	*N*
Holding urine	Never	164
	Sometimes	127
	Frequently	9
Holding defecation	Never	181
	Sometimes	111
	Frequently	8
Postponing meal	Never	131
	Sometimes	157
	Frequently	12
Forgetting or skipping meal	Never	154
	Sometimes	136
	Frequently	10
Facebooking until midnight	Never	53
	Sometimes	202
	Frequently	45

**Table 5:** Intrapersonal factors of Facebook use among respondents (*N* = 300).

Intrapersonal factors	*N*	Percentage (%)
Feel isolated from family		
Agree	109	36.3
Disagree	191	63.7
Feel isolated from society		
Agree	117	39
Disagree	183	61
Room		
Living room	83	27.7
Your bedroom	197	65.7
Dining room	17	5.7
Others	3	1
Refusing to answer calls		
Never	168	56.0
Sometimes	125	41.7
Frequently	7	2.3

**Table 6:** Association between adverse health effects and facebook surfing hours.

Health effects	*N* (%)	Mean rank	Sum of ranks	*P* value
Back pain				
Yes	226 (75.3)	158.13	35737.00	0.006
No	74 (24.7)	127.20	9413.00	
Shoulder/neck pain				
Yes	224 (74.7)	160.27	35901.00	0.001
No	76 (25.3)	121.70	9249.00	
Wrist pain				
Yes	197 (65.7)	157.09	30946.50	0.060
No	103 (34.3)	137.90	14203.50	
Headache				
Yes	207 (69.0)	158.95	32903.00	0.009
No	93 (31.0)	131.69	12247.00	
Eye irritation				
Yes	220 (73.3)	158.25	34814.50	0.008
No	80 (26.7)	129.19	10335.50	

**Table 7:** Association between health related behaviors and facebook surfing hours.

Behaviours	*N* (%)	Mean rank	Sum of ranks	*P* value
Hold urine				
No	164 (54.7)	128.98	21153.00	<0.001
Yes	136 (45.3)	176.45	23997.00	
Hold defecation				
No	181 (60.3)	131.01	23712.00	<0.001
Yes	119 (39.7)	180.15	21438.00	
Postpone meal				
No	131 (43.7)	123.85	16224.00	<0.001
Yes	169 (56.3)	171.16	28926.00	
Forget or skip meal				
No	154 (51.3)	124.94	19241.00	<0.001
Yes	146 (48.7)	177.46	25909.00	
Facebooking until midnight				
No	53 (17.7)	113.89	6036.00	<0.001
Yes	247 (82.3)	158.36	39114.00	

**Table 8:** Association between intrapersonal factors and facebook surfing hours.

Intrapersonal factors	*N* (%)	Mean rank	Sum of ranks	*P* value
Feeling isolated from the family				
Yes	109 (36.3)	174.14	18981.50	<0.001
No	191 (63.7)	137.01	26168.50	
Feeling isolated from the society				
Yes	117 (39.0)	166.47	19476.50	0.008
No	183 (61.0)	140.29	25673.50	
